# Factors associated with child abuse among children and adolescents in a Peruvian public hospital

**DOI:** 10.25122/jml-2022-0262

**Published:** 2023-01

**Authors:** Gabriela Miriam Quille-Mamani, Silvia Cristina Quispe-Prieto, Enaidy Reynosa Navarro

**Affiliations:** 1 Faculty of Health Sciences, Jorge Basadre Grohmann National University, Tacna, Peru; 2 Professional School of Nursing, Faculty of Health Sciences, Jorge Basadre Grohmann National University, Tacna, Peru; 3 Professional School of Medicine, Faculty of Health Sciences, César Vallejo University, Trujillo, Peru

**Keywords:** child abuse, disadvantaged child, disadvantaged adolescent, MAMIS program

## Abstract

The study's objective was to investigate the factors associated with child and adolescent abuse in the MAMIS program at Hipólito Unanue Hospital in the Tacna-Peru region during 2019–2021. The study used a quantitative, retrospective, cross-sectional, and correlational approach to analyze 174 cases of child abuse. The study found that the majority of child abuse cases involved children between the ages of 12–17 (57.4%), with a secondary level of education (51.15%), females (56.9%), and not consuming alcohol or drugs (88.5%). Prevalent household characteristics included single-parent families (48.28%), parents aged 30–59 (58.5%), divorced (37.3%), with secondary level of education (68.9%), independent occupation (64.9%), no history of parental violence (91.3%), no addiction or substance abuse issues (95.4%), and no psychiatric disorders (95.4%). The most common types of abuse were psychological (93.68%), followed by neglect or abandonment (38.51%), physical (37.93%), and sexual (27.0%). The study determined a significant relationship (95% confidence level) between socio-demographic characteristics, such as age, sex, and substance use, and specific types of child abuse.

## INTRODUCTION

Child abuse is a significant global public health issue that encompasses all forms of violence against children under 18, including physical, psychological, and sexual abuse, neglect, negligence, and commercial exploitation. These forms of abuse can harm children and adolescents' health, development, and dignity [1]. Exposure to intimate partner violence is also included among the forms of child abuse. Another factor is that access to essential services such as health care, education, and protection is limited, and the children are at greater risk of falling into trafficking, exploitation, violence, and abuse. Consequently, many children and adolescents worldwide live in environments that are unsafe or conducive to their development. It is estimated that every year, one in two children between the ages of 2 and 17 experiences some form of violence [2]. Around 300 million children between the ages of two and four are often subject to punishment by their caregivers, parents, and other family members [3]. Figures also indicate that children globally experience violence in the form of disciplinary action, usually at home and from an early age [4]. On average, six out of every ten children in the world between the ages of 2 and 14 years suffer periodic physical (bodily) corrections at the hands of their caregivers, where minors are subjected to a mixture of physical punishment and psychological aggression [4, 5].

Peru is not immune to child abuse, and the COVID-19 pandemic has further exposed children and adolescents to situations of abuse. According to the National Aurora Program (MIMP) report, in January 2021 alone, 4,149 cases of child and adolescent violence were reported to the Emergency Center for Women (CEM) in Peru. Children and adolescents aged 12–17 were the most vulnerable group (46.4%), followed by children aged 6–11 (36.6%), and a smaller percentage of children aged 0–5 (16.9%) [6]. This trend was consistent with data from 2020, which showed that 35,661 children and adolescents were treated for violence, with the majority of cases being psychological violence (15,447), followed by physical abuse (10,475), and sexual abuse to a lesser extent. Additionally, there was a 73% increase in sexual harassment cases compared to 2019 [6]. The CEM reported over 1,085,000 cases from 2009 to 2021, with the highest rate of violence in Lima, followed by the Arequipa and Cusco regions at 7.5%, Junín region at 5.8%, and Ancash region at 4.4% [7]. The Tacna region had a lower percentage of reported cases, with 311 complaints of violence against children and adolescents recorded, of which 121 were for psychological violence, 118 for sexual abuse, and a lesser number for physical and sexual violence [8].

The most frequent types of child abuse are physical, emotional, and sexual abuse and acts of omission and physical neglect by parents or other caregivers that trigger damage or threat to the comprehensive development of children and adolescents; these forms can coincide [9, 10]. Child abuse is associated with somatic diseases, emphasizing neurological and musculoskeletal disorders [11]. A study found an association between a history of child abuse and severe premenstrual symptoms in women of childbearing age [12]. The forms of child abuse are increased loneliness, suicidal ideation, depressive states, anxiety, non-suicidal self-harm, increased risk of mental disorders, and decreased coping skills [13–15]. Another study found a significant association between child abuse and the polygenic risk score for schizophrenia, confirming that child abuse is a complex environmental exposure component [16]. Usually, child abuse occurs due to the convergence and simultaneity of factors, which appear mainly at home, with parents and caregivers being the main perpetrators of violence [17].

Some studies consider girls more vulnerable to sexual and psychological abuse while boys are vulnerable to abandonment, with greater emphasis on early childhood, while at older ages, they are vulnerable to physical and sexual abuse [17, 18]. Another vulnerability factor for children and adolescents is the presence of a disability. Children with disabilities are generally at a greater risk for abuse and neglect [17, 19]. Another form of mistreatment, more unusual, is witchcraft, exorcism, and possession; however, there are limited reports and research in this regard [19]. Since the beginning of 2020, the pandemic caused by COVID-19 forced states to implement isolation and confinement measures that had a disruptive impact on family dynamics. In this context, child abuse was drastically exacerbated [20–24].

In Peru, child abuse has become a significant public health issue impacting a significant number of children and adolescents. To address this problem, the Ministry of Health in Peru passed Ministerial Resolution (2011), which included Article 1, approving Health Directive No. 041/MINSA-DGSP-V.01. This directive established guidelines for the operation, management, financing, and implementation of Child and Adolescent Abuse Care Modules (MAMIS) in health facilities across the country. The goal of the MAMIS is to provide comprehensive care and support to children and adolescents affected by abuse [25].

The Child and Adolescent Abuse Care Modules (MAMIS) program is designed as a unit within the Department of Emergency Services, Pediatrics, or the Directorate of the Health Establishment. It consists of a team of professionals and resources, such as human, physical and technological support, organized to provide care for victims of violence, sexual abuse, and exploitation in children and adolescents. The program operates under the principles of providing care based on complexity and resolution capacity levels, providing comprehensive, multidisciplinary care, promoting teamwork, training, and collaborating with other sectors to provide the best possible care for children and adolescents who have experienced abuse [25]. Furthermore, it comprises differentiated spaces where comprehensive and specialized care is provided for the physical and emotional recovery of children and adolescents – from zero to 17 years old – who are victims of different types of abuse (physical, psychological, sexual, abandonment, or negligence) [26]. It generally operates for 12 hours a day and provides care free of charge. It has dedicated and appropriate facilities, as well as a team of qualified professionals, including a psychologist, psychiatrist, pediatrician, gynecologist, obstetrician, nurse, social worker, and lawyer. This team enables timely intervention for the physical and emotional recovery of girls, boys, and adolescents who are victims of violence [26, 27].

The objective of this study was to determine the factors associated with the type of child and adolescent abuse in the MAMIS program, Hipólito Unanue hospital, Tacna-Peru region, 2019–2021.

## MATERIAL AND METHODS

### Design of the investigation

The research adopts a positivist paradigm and employs a non-experimental, observational, correlational, and retrospective design to collect data on cases of abuse in the Tacna region of Peru between 2019 and 2021.

### Population and sample

The population for this study consisted of 174 medical records of children and adolescents identified as victims of child abuse by the MAMIS program at Hipólito Unanue Hospital in the Tacna region of Peru between 2019 and 2021. All cases were confirmed through evidence in the medical history of the individuals.

### Data collection and analysis

The research employed a combination of observation and survey techniques to collect data on socio-demographics, household characteristics, and child abuse. The first instrument was the data collection form focused on socio-demographics and household characteristics [28], modified by Quille [29], which includes 13 items structured into two parts: personal aspects (5 items) and family aspects (8 items). The reliability of this instrument was determined using Cronbach's alpha coefficient score of 0.74. To measure the child abuse variable and to classify violence in four dimensions, the "Violence Screening of the Ministry of Health of Peru", modified by Quille, was used [29]. The instrument consists of 33 items, physical abuse (16 items) psychological abuse (16 itemms), sexual abuse (4 items), and neglect or abandonment (6 items). The reliability of this instrument was determined using the Cronbach's alpha coefficient score of 0.72.

The data was collected from medical records of children and adolescents treated by the MAMIS program at Hipólito Unanue hospital in the Tacna region of Peru between 2019 and 2021. The instruments were filled out individually, and the answers were coded and processed using Microsoft Excel and statistical software SPSS V.27.0. The research data was then grouped and used to create figures and summaries, with results presented by variable, dimension, and indicator.

### Ethical considerations

All the information was collected from the medical records of children and adolescents treated by the MAMIS program, Hipólito Unanue hospital, Tacna-Peru region, 2019–2021, through a data collection form on children and adolescents in the program of the Module of Attention to Child Abuse of Children and Adolescents in Health (MAMIS) in the hospital and the indicated dates. The ethical principles in the research were taken into account, which is the following: protection of people, beneficence and non-maleficence, justice, and scientific integrity [30].

## RESULTS

Table 1 indicates that the majority of child abuse cases (41.38%) occurred outside the districts included in the survey. The district of Gregorio Albarracín had the second-highest rate at 23.51%. The age group most affected was 12–17 years, making up 57.47% of cases. The next most affected age group was 6–11 years, accounting for 27.01% of cases. The majority of victims (51.15%) had a secondary level of education. Female children and adolescents were the most affected, comprising 56.9% of cases. Importantly, 88.5% of victims did not consume alcohol or drugs.

**Table 1:** Socio-demographic characteristics of children and adolescents, MAMIS program, Hipólito Unanue hospital, Tacna-Peru region, 2019–2021.

Socio-demographic characteristics of children		
	N	%
**Child age**		
0–11 months	3	1.72
1–5 years	24	13.79
6–11 years	47	27.01
12–17 years	100	57.47
Total	**174**	**100.0**
**Sex**		
Female	99	56.90
Male	75	43.10
Total	**174**	**100.0**
**District**		
New city	25	14.37
High of the Alliance	36	20.69
Gregorio Albarracin	41	23.56
Others	72	41.38
Total	**174**	**100.0**
**Level of education**		
Preschool	18	10.34
Primary	64	36.78
Secondary	89	51.15
None	3	1.72
Total	**174**	**100**
**Alcohol and/or drug use**		
Yes	20	11.49
No	154	88.51
Total	174	100.0

The most common type of family structure was single-parent (48.28%). The majority of mothers (51.72%) and fathers (58.08%) were between the ages of 30–59 years. Most parents were divorced (37.36%), or cohabiting (26.44%). The educational level of parents was predominantly secondary level, with 28.97% for mothers and 65.52% for fathers. The majority of parents had independent occupations, representing 64.94% for mothers and 42.10% for fathers. Notably, there was no history of violence among 91.38% of parents, and 83.33% did not have a history of addiction or drug use. Additionally, 95.40% of parents did not have psychiatric disorders (Table 2).

**Table 2:** Household characteristics of children and adolescents, MAMIS program, Hipólito Unanue hospital, Tacna-Peru region, 2019–2021.

Household characteristics		
	N	%
**Type of family**		
Nuclear	55	31.61
Extensive	35	20.11
Single parent	84	48.28
Total	**174**	**100.0**
**Mother's age**		
Teenager (12–17 years old)	7	4.02
Young (18–29 years old)	77	44.25
Adult (30–59 years)	90	51.72
Total	**174**	**100.0**
**Father's age**		
Teenager (12–17 years old)	two	1.15
Young (18–29 years old)	71	40.80
Adult (30–59 years)	101	58.05
Total	174	100.0
**Marital status**		
Single	42	24.14
Married	16	9.20
Divorced	65	37.36
Cohabitant	46	26.44
Other	5	2.87
Total	174	100.0
**Mother's level of education**		
Primary	9	5.17
Secondary	120	68.97
Higher	35	20.11
None	10	5.75
Total	174	100.0
**Father's educational level**		
Primary	0	0.00
Secondary	114	65.52
Higher	52	29.89
None	8	4.60
Total	174	100
**Mother's occupation**		
Student	5	2.87
Worker	5	2.87
Employee	12	6.90
Independent	113	64.94
Other	39	22.41
Total	174	100
**Father's occupation**		
Student	4	2.30
Worker	37	21.26
Employee	19	10.92
Independent	75	43.10
Other	39	22.41
Total	174	100
**History of suffered violence**		
Yes	fifteen	8.62
No	159	91.38
Total	174	100
**Addiction or consumption of drugs and/or alcohol**		
Yes	29	16.67
No	145	83.33
Total	174	100
**Presence of psychiatric disorders**		
Yes	8	4.60
No	166	95.40
Total	174	100

Table 3 presents the relationship between the type of child abuse and socio-demographic characteristics among children and adolescents. The highest percentage of abuse (54%) was found in the age factor, with ages between 12 and 17 years most commonly affected by psychological abuse. Additionally, 54% of female children and adolescents present psychological abuse. Regarding the level of education, 47.1% of those affected by psychological abuse were in secondary school. We identified significant relationships between age and psychological abuse (p=0.000) and sexual abuse (p=0.007); between gender and type of physical abuse (P=0.019), sexual abuse (P=0.000) and abandonment and neglect (P=0.025); between educational level and physical abuse (P=0.004) and sexual abuse (P=0.037). No association was found between the place of residence of the child or adolescent or the parents' alcohol/drug consumption.

**Table 3:** The relationship between socio-demographics and child abuse at MAMIS, Hipólito Unanue hospital, Tacna region-Peru, 2019–2021.

Socio-demographics	Child abuse																	
	Physical				Psychological				Sexual abuse				Abandonment or neglect				Total	
**Child age**	A	%	P	%	A	%	P	%	A	%	P	%	A	%	P	%	T	%
0–11 months	two	1.1	1	0.6	two	1.1	1	0.6	3	1.7	0	0.0	1.0	0.6	2.0	1.1	3	1.7
1–5 years	13	7.5	eleven	6.3	3	1.7	twenty-one	12.1	twenty-one	12.1	3	1.7	12.0	6.9	12.0	6.9	24	13.8
6–11 years	35	20.1	12	6.9	0	0.0	47	27.0	40	23.0	7	4.0	29.0	16.7	18.0	10.3	47	27.0
12–17 years	58	33.3	42	**24.1**	6	3.4	94	**54.0**	63	36.2	37	**21.3**	65.0	37.4	35.0	**20.1**	100	**57.5**
TOTAL	108	62.1	66	37.9	eleven	6.3	163	93.7	127	73.0	47	27.0	107.0	61.5	67.0	38.5	174	100.0
x2	P=0.218>0.05				**P=0.000<0.05**				**P=0.007<0.05**				P=0.413>0.05				-	
**Sex**	A	%	P	%	A	%	P	%	A	%	P	%	A	%	P	%	T	%
Feminine	54	31.0	Four. Five	**25.9**	5	2.9	94	**54.0**	62	35.6	37	**21.3**	68	39.1	31	17.8	99	56.9
Male	54	31.0	twenty-one	12.1	6	3.4	69	39.7	65	37.4	10	5.7	39	22.4	36	**20.7**	75	43.1
TOTAL	108	62.1	66	37.9	eleven	6.3	163	93.7	127	73.0	47	27.0	107	61.5	67	38.5	174	100.0
x2	**P=0.019<0.05**				P=0.429>0.05				**P=0.000<0.05**				**P=0.025<0.05**				-	
**District**	A	%	P	%	A	%	P	%	A	%	P	%	A	%	P	%	T	%
New city	13	7.5	12	6.9	4	23	twenty-one	12.1	twenty-one	12.1	4	23	16	9.2	9	5.2	25	14.4
High of the Alliance	25	14.4	eleven	6.3	two	1.1	3. 4	19.5	25	14.4	eleven	6.3	22	12.6	14	8.0	36	20.7
Gregorio Albarracin	23	13.2	18	10.3	3	1.7	38	21.8	29	16.7	12	6.9	30	17.2	eleven	6.3	41	23.6
Others	47	27.0	25	**14.4**	two	1.1	70	**40.2**	52	29.9	twenty	**11.5**	39	22.4	33	**19.0**	72	41.4
x2	P=0.416>0.05				P=0.134>0.05				P=0.595>0.05				P=0.255>0.05				-	
**Level of education**	A	%	P	%	A	%	P	%	A	%	P	%	A	%	P	%	T	%
Preschool	6	3.4	12	6.9	3	1.7	fifteen	8.6	16	9.2	two	1.1	eleven	6.3	7	4.0	18	10.3
Primary	48	27.6	16	9.2	1	0.6	63	36.2	51	29.3	13	7.5	37	21.3	27	15.5	64	36.8
Secondary	51	29.3	38	21.8	7	4.0	82	**47.1**	57	32.8	32	**18.4**	59	33.9	30	**17.2**	89	51.1
None	3	1.7	0	0.0	0	0.0	3	1.7	3	1.7	0	0.0	0	0.0	3	1.7	3	1.7
x2	**P=0.004<0.05**				P=0.100>0.05				**P=0.037<0.05**				P=0.110>0.05				-	
**Alcohol/drug use**	A	%	P	%	A	%	P	%	A	%	P	%	A	%	P	%	T	%
Yes	8	4.6	12	6.9	1	0.6	19	10.9	18	10.3	two	1.1	14	8.0	6	3.4	twenty	11.5
No	100	57.5	54	**31.0**	10	5.7	144	**82.8**	109	62.6	Four. Five	**25.9**	93	53.4	61	**35.1**	154	88.5
TOTAL	108	62.1	66	37.9	eleven	6.3	163	93.7	127	73.0	47	27.0	107	61.5	67	38.5	174	100.0
	**P=0.031<0.05**				P=0.796>0.05				P=0.069>0.06				P=0.406>0.06				-	

Table 4 presents the relationship between household characteristics and type of child abuse among children and adolescents. The highest percentage of child abuse (44.3%) occurred in single-parent families and was primarily psychological abuse. The age of both parents was also a significant factor, with the highest abuse occurring in adults (30–59%) and being psychological. Regarding marital status, divorced parents had the highest percentage of abuse (36%), with psychological abuse being the most common form. Mothers with a secondary level of education had the highest percentage of physical abuse (64.4%), while fathers with a secondary level of education had the highest percentage of psychological abuse (59.2%). The highest percentage of independent occupation and psychological abuse falls at 61.5% (among females) and 39% (males). The Chi-square test was applied to determine the association between variables, and there was an association between the mother's age and sexual abuse (P=0.017) and between the father's level of education and the type of psychological abuse (P=0.045). No association was found for other variables.

**Table 4:** Relationship between household characteristics and child abuse among children and adolescents.

Household characteristics	Child abuse																	
	Physical				Psychological				Sexual abuse				Abandonment or neglect				Total	
**Type of family**	A	%	P	%	A	%	P	%	A	%	P	%	A	%	P	%	T	%
Nuclear	35	20.1	twenty	11.5	two	1.1	53	30.5	43	24.7	12	6.9	32	18.4	23	13.2	55	31.6
Extensive	19	10.9	16	9.2	two	1.1	33	19.0	25	14.4	10	5.7	17	9.8	18	10.3	35	20.1
Single parent	54	31.0	30	**17.2**	7	4.0	77	**44.3**	59	33.9	25	**14.4**	58	33.3	26	**14.9**	84	48.3
Total	108	62.1	66	37.9	eleven	6.3	163	93.7	127	73.0	47	27.0	107	61.5	67	38.5	174	100.0
P<0.05	P=0.567>0.05				P=0.531>0.05				P=0.572>0.05				P=0.093>0.05				-	
**Mother's age**	A	%	P	%	A	%	P	%	A	%	P	%	A	%	P	%	T	%
Teenager (12–17 years old)	4	23	3	1.7	1	0.6	6	3.4	7	4.0	0	0.0	3	1.7	4	23	7	4.0
Young (18–29 years old)	54	31.0	23	13.2	6	3.4	71	40.8	62	35.6	fifteen	8.6	46	26.4	31	17.8	77	44.3
Adult (30–59 years)	fifty	28.7	40	**23.0**	4	23	86	**49.4**	58	33.3	32	**18.4**	58	33.3	32	**18.4**	90	51.7
Total	108	62.1	66	37.9	eleven	6.3	163	93.7	127	73.0	47	27.0	107	61.5	67	38.5	174	100.0
P<0.05	P=0.148>0.05				P=0.457>0.05				**P=0.017<0.05**				P=0.482>0.05				-	
**Father's age**	A	%	P	%	A	%	P	%	A	%	P	%	A	%	P	%	T	%
Teenager (12–17 years old)	two	1.1	0	0.0	0	0.0	two	1.1	two	1.1	0	0.0	0	0.0	two	1	two	1
Young (18–29 years old)	Four. Five	25.9	26	14.9	6	3.4	65	37.4	58	33.3	13	7.5	43	24.7	28	16	71	41
Adult (30–9 years)	61	35.1	40	**23.0**	5	2.9	96	**55.2**	67	38.5	3. 4	**19.5**	64	36.8	37	**twenty-one**	101	58
Total	108	62.1	66	37.9	eleven	6.3	163	93.7	127	73.0	47	27.0	107	61.5	67	39	174	100
P<0.05	P=0.498>0.05				P=0.607>0.05				P=0.057>0.05				P=0.185>0.05				-	
**Marital status**	A	%	P	%	A	%	P	%	A	%	P	%	A	%	P	%	T	%
Single	28	16.1	14	8	two	1	40	23	35	twenty	7	4	24	14	18	10	42	24
Married	10	5.7	6	3	1	1	fifteen	9	10	6	6	3	12	7	4	two	16	9
Divorced	38	21.8	27	**16**	3	two	62	**36**	46	26	19	**eleven**	Four. Five	26	twenty	eleven	65	37
Cohabitant	28	16.1	18	10	5	3	41	24	31	18	fifteen	9	24	14	22	**13**	46	26
Others	4	23	1	1	0	0	5	3	5	3	0	0	two	1	3	two	5	3
Total	108	62.1	66	38	eleven	6	163	94	127	73	47	27	107	61	67	39	174	100
P<0.05	P=0.836>0.05				P=0.656>0.05				P=0.205>0.05				P=0.209>0.05				-	
**Educational level (mother)**	A	%	P	%	A	%	P	%	A	%	P	%	A	%	P	%	T	%
Primary	two	1.1	7	4.0	5	2.9	4	23	7	4.0	two	1.1	7	4.0	two	1.1	9	5.2
Secondary	8	4.6	112	**64.4**	74	42.5	46	**26.4**	87	50.0	33	**19.0**	73	42.0	47	**27.0**	120	69.0
Higher	0	0.0	35	20.1	23	13.2	12	6.9	24	13.8	eleven	6.3	twenty	11.5	fifteen	8.6	35	20.1
None	1	0.6	9	5.2	6	3.4	4	23	9	5.2	1	1	7	4.0	3	1.7	10	5.7
Total	eleven	6.3	163	93.7	108	62.1	66	37.9	127	73.0	47	27.0	107	61.5	67	38.5	174	100.0
P<0.05	P=0.091>0.05				P=0.943>0.05				P=0.586>0.05				P=0.656>0.05				-	
**Educational level (dad)**	A	%	P	%	A	%	P	%	A	%	P	%	A	%	P	%	T	%
Primary	0	0.0	0	0.0	0	0.0	0	0.0	0	0.0	0	0.0	0	0.0	0	0.0	0	0.0
Secondary	69	39.7	Four. Five	**25.9**	eleven	6.3	103	**59.2**	83	47.7	31	**17.8**	70	40.2	44	**25.3**	114	65.5
Higher	33	19.0	19	10.9	0	0.0	52	29.9	36	20.7	16	9.2	32	18.4	twenty	11.5	52	29.9
None	6	3.4	two	1.1	0	0.0	8	4.6	8	4.6	0	0.0	5	2.9	3	1.7	8	4.6
Total	108	62.1	66	37.9	eleven	6.3	163	93.7	127	73.0	47	27.0	107	61.5	67	385	174	100.0
P<0.05	P=0.696>0.05				**P=0.045<0.05**				P=0.189>0.05				P=0.998>0.05				-	
**Mother's occupation**	A	%	P	%	A	%	P	%	A	%	P	%	A	%	P	%	T	%
Student	5	2.9	0	0.0	0	0.0	5	2.9	5	2.9	0	0.0	1	0.6	4	23	5	12.8
Worker	3	1.7	two	1.1	0	0.0	5	2.9	4	23	1	0.6	3	1.7	two	1.1	5	2.9
Employee	9	5.2	3	1.7	two	1.1	10	5.7	8	4.6	4	23	10	5.7	two	1.1	12	6.9
Independent	66	37.9	47	**27.0**	6	3.4	107	**61.5**	79	45.4	3. 4	**19.5**	68	39.1	Four. Five	**25.9**	113	64.9
Other	25	14.4	14	8.0	3	1.7	36	20.7	31	17.8	8	4.6	25	14.4	14	8.0	39	22.4
Total	108	62.1	66	37.9	eleven	6.3	163	93.7	127	73.0	47	27.0	107	61.5	67	38.5	174	100.0
P<0.05	P=0.328>0.05				P=0.531>0.05				P=0.463>0.05				P=0.181>0.05				-	
**Father's occupation**	A	%	P	%	A	%	P	%	A	%	P	%	A	%	P	%	T	%
Student	3	1.7	1	0.6	0	0	4	23	4	23	0	0	two	1.1	two	1.1	4	10
Worker	twenty	12	17	9.8	0	0	37	twenty-one	22	13	fifteen	8.6	18	10	19	eleven	37	twenty-one
Employee	12	6.9	7	4	1	0.6	18	10	fifteen	8.6	4	23	17	9.8	two	1.1	19	eleven
Independent	42	24	33	**19**	8	4.6	67	**39**	55	32	twenty	**12**	54	31	twenty-one	12	75	43
Other	31	18	8	4.6	two	1.1	37	twenty-one	31	18	8	4.6	16	9.2	23	**13**	39	22
Total	108	62	66	38	eleven	6.3	163	94	127	73	47	27	107	62	67	39	174	100
P<0.05	P=0.112>0.05				P=0.259>0.05				P=0.192>0.05				**P=0.001<0.05**				-	
**History of violence**	A	%	P	%	A	%	P	%	A	%	P	%	A	%	P	%	T	%
Yes	9	5.2	6	3.4	1	0.6	14	8	eleven	6.3	4	23	10	5.7	5	2.9	fifteen	8.6
No	99	57	60	**35**	10	5.7	149	**86**	116	67	43	**25**	97	56	62	**36**	159	91
Total	108	62	66	38	eleven	6.3	163	94	127	73	47	27	107	62	67	39	174	100
P<0.05	P=0.863>0.05				P=0.954>0.05				P=0.975>0.05				P=0.667>0.05				-	
**Addiction or consumption**	A	%	P	%	A	%	P	%	A	%	P	%	A	%	P	%	T	%
Yes	18	10	eleven	6.3	3	1.7	26	fifteen	23	13	6	3.4	14	8	fifteen	8.6	29	17
Nope	90	52	55	**32**	8	4.6	137	**79**	104	60	41	**24**	93	53	52	**30**	145	83
Total	108	62	66	38	eleven	6.3	163	94	127	73	47	27	107	62	67	39	174	100
P<0.05	P=1.000>0.05				P=0.329>0.05				P=0.401>0.05				P=0.109>0.05				-	
**Psychiatric disorders**	A	%	P	%	A	%	P	%	A	%	P	%	A	%	P	%	T	%
Yes	6	3.4	two	1.1	1	0.6	7	4	7	4	1	0.6	3	1.7	5	2.9	8	4.6
No	102	59	64	**37**	10	5.7	156	**90**	120	69	46	**26**	104	60	62	**36**	166	95
Total	108	62	66	38	eleven	6.3	163	94	127	73	47	27	107	62	67	39	174	100
P<0.05	P=0.440>0.05				P=0.462>0.05				P=0.344>0.05				P=0.153>0.05				-	

## DISCUSSION

This study was conducted at the Child and Adolescent Abuse Care Module in Health at the Hipólito Unanue Hospital in Tacna, Peru. 174 cases of child abuse in children and adolescents were analyzed to determine the factors associated with the type of child abuse. The main objective of the study was to identify the factors associated with the type of child and adolescent abuse in the MAMIS program at the Hipólito Unanue Hospital in the Tacna region of Peru between 2019–2021.

Child abuse can occur in urban and rural areas, demonstrating that this type of abuse manifests itself in different spaces, places, and forms. This investigation found that the highest percentage of child abuse cases in the Tacna-Peru region occurred in districts located away from the main city of Tacna. This is likely due to cultural customs that involve the use of physical punishment as a disciplinary method, which is often perceived as normal. Additionally, parents' frustrations related to economic and cultural factors may also contribute to an increase in child abuse cases. The study revealed that the age group most affected by child abuse was 12–17 years old, with the majority of victims having a secondary education level. Adolescence is a particularly challenging stage for both young people and parents, and this stage is characterized by significant physical and emotional changes. The majority of victims were females, and the majority did not consume alcohol or drugs.

Pedroso (2021) highlights that gender and stage of development are key factors that can increase a child's susceptibility to abuse. Gender inequality can increase girls' risks of abuse, violent relationships, or exploitation, and the widespread use of violence within the family and society to deal with conflict [17]. Therefore, it is agreed that violence against children and adolescents hurts child development, impacting minors' physical, mental and social health [31]. The study by Salcedo (2019) shows similar data where children under ten years of age are at the optimal age for schooling but may prioritize play over educational or household tasks. This situation can lead to child abuse when their parents are unaware of or ignore this reality [32].

Similarly, Salmon's (2022) study of adolescents aged 14 to 17 found that abuse occurs more frequently among males during this stage of development. This is likely related to the psychological and physical changes that occur during adolescence. The study found that males are more likely to be victims of child abuse than females. González-Sábado *et al*. (2019) confirm these findings, with the group of males experiencing the highest rate of violence at 58.06%. The age group with the highest rate of violence was 12–14 years (35.48%), followed by 9–11 years (25.80%). This shows that the risk of violence increases with age, with most cases occurring in children over nine. These studies demonstrate that both the child's gender and stage of development can play a role in determining their susceptibility to abuse.

According to the MAMIS program, household characteristics that contribute to child abuse among children and adolescents include the type of single-parent family where the mother is primarily responsible for the care of the children. This situation can lead to a negligent parenting style, excessive permissiveness, and the absence of a father figure, which can put children at risk for abuse [33]. The predominant marital status among the parents is divorced (37.36%), sometimes presenting family violence. For this reason, the family must be the main pillar for children and adolescents to build their personality, principles, and moral obligations and develop the essential capacities to solve complex problems.

Most of the parents in the study had a secondary level of education (28.97%). Many of them (64.94%) had independent occupations. Parents with higher levels of education tend to create a safer and more nurturing environment for their children as they have a better understanding of how to educate and discipline their children without resorting to physical or psychological punishment. However, the demands of their occupation can sometimes lead to neglect of their children. 42.10% of the parents in the study did not report having suffered violence. Additionally, 91.38% of the parents reported no addiction or drug use problems, and 95.40% reported no psychiatric disorders.

It is well-established that the various forms of child abuse perpetrated by parents or other caregivers harm the overall development of children and adolescents [34–36]. Likewise, associations are found with poor cognitive abilities or performance in childhood and even into adulthood, especially with specific types of abuse that will lead to the loss of opportunities and limited personal development [13, 14]. The results are similar to the research by García-Cruz *et al*., where the majority of single-parent families had young mothers (47.2%), primary caregivers (68%), and single mothers (47.2%) (83, 3%) [37]. In contrast, the study by Salcedo D. differs from this study where the type of family, 60.7% belong to a nuclear family, 29.5% to an extended family, and 9.8% to a single-parent family. This study highlights that nuclear families made up of a couple and their children, tend to have fewer instances of violence as both parents are actively involved in caring for and raising their children [32].

We found an association between age and the type of psychological abuse and sexual abuse (p<0.05). Other significant associations were found between gender and type of physical abuse, sexual abuse, abandonment and neglect (p<0.05), educational level, and physical abuse and sexual abuse (p>0.05). No association was found between the place of residence and alcohol/drug use by parents. These findings are consistent with Solis G. *et al*., who mention that sexual abuse was more frequent in girls and physical abuse more common in boys. It was also observed that the probability of hospitalization is greater in children under one year of age due to violence, decreases as age increases, and changes with the type of abuse. Leppäkoski, in his study, identified that 43.9% of four-year-old children experienced at least one form of psychological abuse by their parents or some other close person and a lower percentage of physical abuse. Psychological and physical abuse were also shown to have occurred simultaneously. In contrast, physical abuse predominated among boys compared to girls and children of Finnish origin compared to children of foreign origin [10]. Like Guerrero D *et al*., a predominance of female patients (65.8%) was observed, where physical abuse was the most frequent type of abuse among patients with a final diagnosis of child abuse admitted through the emergency service in Mexico [38].

We identified a significant association between the mother's age and sexual abuse and the father's educational level and type of psychological abuse (P<0.05) (Table 4). Other variables did not show a significant association. These findings are consistent with a study that has identified factors such as economic situation and family income, unemployment, the number of household members, unwanted children, affective preference for certain children, history of abuse and violence, marital differences, separation or divorce, illness, disabilities, and children's learning problems, as potential contributors to child abuse [39]. The type of violence was negatively associated with the age difference between the mother and her spouse, the mother's education, and the level of happiness in the home. All the relationships discovered are consistent with what is found in the literature, implying that the homes where the mother has a history of physical violence are also the homes where the boys and girls suffer physical violence. These findings support the results of the present study because a home with a history of domestic violence drastically impairs the development of children and adolescents [32]. Also, the study by Robledo M. *et al*. found that child abuse was the most frequent complication, followed by abandonment, and in most cases, the abuser was a relative or acquaintance of the child. Being a teenage mother, having a low educational level, having a low income, illicit drug use, having a child with a disability, and being under two years of age were the highest risk factors for child abuse [40].

Adequate communication channels within the family are crucial in addressing and preventing child abuse at home. The study suggests that lack of communication can lead to psychological abuse and that good coexistence does not necessarily depend on the level of education of the aggressor. The occupation of both parents and any changes in employment can directly impact the likelihood of psychological abuse, neglect, or abandonment of children and adolescents. This is because when parents are away from home, they may be stressed and worry about their livelihood, which can lead to unloading frustration, oppression, and neglect on their children, resulting in psychological abuse and neglect [33, 41].

In this study, parents with a history of violence, addiction, or alcohol consumption were not found to be more likely to abuse their children, unlike Lawson *et al*., who found that parents with a history of psychological abuse were more likely to continue psychological abuse during the pandemic than those without a history of psychological abuse before the pandemic [42]. This study also found that as parental depressive symptoms increased in the wake of COVID-19, so did the odds of psychologically abusing children during the pandemic.

## CONCLUSIONS

Gender gaps can increase the risks of violence and exploitation for girls, especially in adolescence, that include early sexual relations, sexual exploitation, assuming household roles that limit their time for leisure and study, and manifestations of masculinity and family violence that may occur within the family. In the case of single-parent families, there is the risk of negligent educational style, characterized by excessive permissiveness, which directly affects the overall happiness of children and adolescents. These issues emphasize the importance of the family as a fundamental pillar where children and adolescents can find healthy spaces to build thaving heir personalities through self-knowledge, values, self-esteem, resilience, and other socio-emotional factors. Economic challenges such as unemployment or overwork, when parents prioritize work over family time, can also lead to abandonment or psychological abuse in children and adolescents. Child abuse, including physical, psychological, and sexual abuse, as well as neglect, is often associated with demographics and household characteristics in the study population, consistent with other contexts, highlighting that the older the child and adolescent, the more likely these forms of abuse will occur. Finally, rural contexts should also be considered, where some parents use physical discipline to the detriment of children's physical, mental and social health.

## Data Availability

The data analyzed in this research is available in Open Access on the Zenodo platform under the terms of the Creative Commons Attribution 4.0 International license (CC-BY 4.0).
